# Cellular Origins of EGFR‐Driven Lung Cancer Cells Determine Sensitivity to Therapy

**DOI:** 10.1002/advs.202101999

**Published:** 2021-10-07

**Authors:** Fan Chen, Jinpeng Liu, Robert M. Flight, Kassandra J. Naughton, Alexsandr Lukyanchuk, Abigail R. Edgin, Xiulong Song, Haikuo Zhang, Kwok‐Kin Wong, Hunter N. B. Moseley, Chi Wang, Christine F. Brainson

**Affiliations:** ^1^ Department of Toxicology and Cancer Biology University of Kentucky Lexington KY 40536 USA; ^2^ Department of Internal Medicine University of Kentucky Lexington KY 40536 USA; ^3^ Department of Molecular and Cellular Biochemistry University of Kentucky Lexington KY 40536 USA; ^4^ Markey Cancer Center University of Kentucky Lexington KY 40536 USA; ^5^ DNAtrix 10355 Science Center Drive, Suite 110 San Diego CA 92121 USA; ^6^ Laura and Isaac Perlmutter Cancer Center NYU Langone Medical Center New York University New York NY 10016 USA; ^7^ Present address: Department of Medical Oncology Sun Yat‐sen University Cancer Center State Key Laboratory of Oncology in South China Collaborative Innovation Center for Cancer Medicine Sun Yat‐sen University Guangzhou 510060 P. R. China

**Keywords:** alveolar, bronchiolar, EGFR, lung cancer, organoids

## Abstract

Targeting the epidermal growth factor receptor (EGFR) with tyrosine kinase inhibitors (TKIs) is one of the major precision medicine treatment options for lung adenocarcinoma. Due to common development of drug resistance to first‐ and second‐generation TKIs, third‐generation inhibitors, including osimertinib and rociletinib, have been developed. A model of EGFR‐driven lung cancer and a method to develop tumors of distinct epigenetic states through 3D organotypic cultures are described here. It is discovered that activation of the *EGFR T790M/L858R* mutation in lung epithelial cells can drive lung cancers with alveolar or bronchiolar features, which can originate from alveolar type 2 (AT2) cells or bronchioalveolar stem cells, but not basal cells or club cells of the trachea. It is also demonstrated that these clones are able to retain their epigenetic differences through passaging orthotopically in mice and crucially that they have distinct drug vulnerabilities. This work serves as a blueprint for exploring how epigenetics can be used to stratify patients for precision medicine decisions.

## Graphical abstract

In the report, murine lung organoids derived from distinct lung epithelial cells are utilized for transformation via oncogenic epidermal growth factor receptor (EGFR). The findings show that EGFR has differential oncogenic capacity in distinct lung cells and that lung cell lineage identity influences response to therapies.

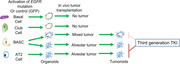

## 1 Introduction

Understanding the cellular and molecular origins of lung cancer will help us to define ways to prevent this deadly disease. Different stem or progenitor cells of adult lung reside throughout the trachea to the distal alveoli, including basal cells and club cells in the proximal airways as well as bronchioalveolar stem cells (BASCs) and alveolar type 2 (AT2) cells in the distal airways.^[^ ^1^, ^2^, ^3^ ^]^ Lung adenocarcinoma has been postulated to originate from club cells, AT2 cells, or BASCs, while squamous cell carcinomas likely arise from basal cells.^[^ ^4^, ^5^, ^6^ ^]^ Experimental models have shown that given different oncogenes, lung cells can be more or less “fit” to form fully malignant lesions.^[^ ^7^, ^8^, ^9^ ^]^ However, the cellular origins for the majority of lung cancers have not been clarified yet. Determining common lung tumor origins may help us to understand how to prevent malignant transformation and guide us to using appropriate therapeutics.

Precision medicine options for lung cancer have greatly expanded in the past decade. Biomarkers encoded by genetic changes are currently the predominant tools for deciding precision medicine options. However, epigenetic biomarkers, including markers of cell states, could add crucial additional predictions of drug responses. As an example, for lung adenocarcinoma there are at least three distinct reproducible subtypes that have been identified through transcriptomics approaches, and each of these subtypes may have not only genetic but also epigenetic determinants of drug response.^[^ ^10^ ^]^ These three subtypes have been termed bronchoid, magnoid, and squamoid. From the perspective of lung epithelial cell types, squamoid may represent bronchiolar epithelial cells including SOX2+ secretory club cells or basal cells, while bronchoid may represent more distal alveolar type 2 cells. Also, bronchoid tumors tend to be more acinar and better prognosis, while squamoid have more solid pattern and worse prognosis. Magnoid tumors are characterized by high cell cycle and poorer prognosis. Transcriptionally distinct subtypes may provide an additional layer of guidance for prognosis or therapeutics in clinics.

One of the most successful precision medicine options for lung cancer patients is targeting the epidermal growth factor receptor (EGFR) with drugs that inhibit the tyrosine kinase function of the activated protein.^[^ ^11^ ^]^ Gefitinib was the first EGFR inhibitor, followed quickly by erlotinib, but both inhibitors were plagued by the development of drug resistance, in many cases through mutation of the T790M gatekeeper residue of EGFR.^[^ ^12^, ^13^ ^]^ Second generation irreversible and third generation mutant‐selective inhibitors including afatinib, osimertinib, and rociletinib were developed.^[^ ^14^ ^]^ Most recently, the third generation tyrosine kinase inhibitor (TKI) osimertinib has been moved to a first line agent for EGFR mutant lung cancer.^[^ ^15^ ^]^ However, acquired resistance to osimertinib has also emerged in patients potentially resulting from EGFR mutations (C797S, G724S, and L718Q), loss of T790M, pathway bypass through KRAS proto‐oncogene, GTPase (KRAS), MET proto‐oncogene, receptor tyrosine kinase (MET), or PIK3CA activation, or small‐cell lung cancer transformation.^[^ ^16^, ^17^, ^18^ ^]^

Here we describe a model of EGFR‐driven lung cancer and a method to develop tumors of distinct epigenetic states through the use of 3D organotypic cultures. We discovered that EGFR mutation led to lung cancer with alveolar or bronchiolar features, which can originate from AT2 cells or BASCs, but not basal cells or club cells of the trachea. We also demonstrate that these clones were able to retain their epigenetic differences through passaging orthotopically in mice and crucially that they had distinct drug vulnerabilities that can be modulated through drug combinations. This work serves as a blueprint for exploring how epigenetics can be used to stratify patients for precision medicine decisions.

## 2 Results and Discussion

### 2.1 The Autochthonous lox‐stop‐lox: EGFR T790M/L858R Model Develops Lung Adenocarcinoma in Mice

**Figure 1:**
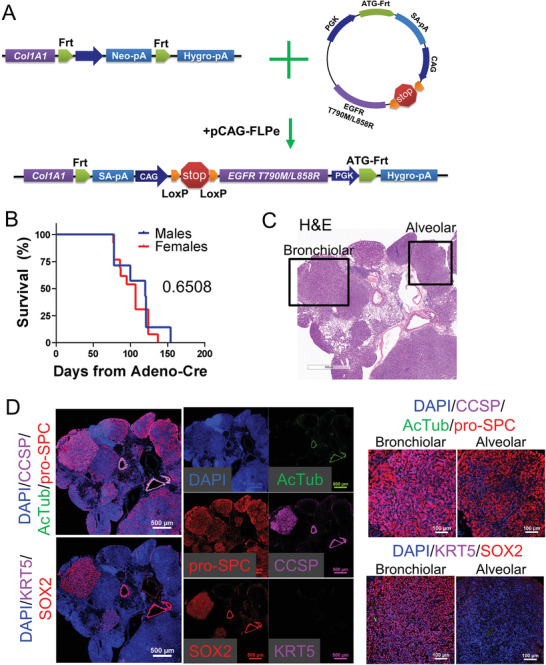
The autochthonous LSL:EGFR T790M/L858R model develops lung adenocarcinoma in mice. A) Schematic of mouse model with LoxP‐mediated activation of EGFR T790M/L858R mutations. B) Overall survival of mice of the indicated gender is graphed, *n* = 7 male, *n* = 13 female. C) Representative H&E stained cross sections from lungs of EGFR T790M/L858R autochthonous model. D) Representative immunofluorescence staining of EGFR T790M/L858R autochthonous lung tumors with the indicated probes.

To interrogate the cell‐of‐origin for EGFR‐driven lung cancer,we utilized a model generated by knocking in the human *EGFR* gene encoding protein with both T790M and L858R mutations (*EGFR* TL) into the *collagen1a1* (*Col1a1*) locus (**Figure** **1**A). The EGFR TL mutations were used in the model because patients usually had L858R mutation first and acquired the second T790M mutation to become resistant to first or second generation TKIs. The resulting mice contain one *Col1a1* locus that had been replaced with a lox‐stop‐lox (LSL) cassette followed by the cytomegalovirus, chicken beta‐actin, rabbit beta‐globin (CAG) promoter and a mutant EGFR gene. Cre‐mediated deletion of the lox‐stop‐lox site was accomplished by intranasal instillation of an adeno‐Cre virus, which others have shown to infect random cells from the proximal to distal airways.^[^ ^19^, ^20^, ^21^ ^]^ The median of the overall survival in this model was 107 d, 120 d for males and 107 d for females with no statistically significant difference between the males and females (*p *= 0.65, Figure 1B). The tumors that formed by intranasal adeno‐Cre installation were almost exclusively alveolar type adenocarcinoma, with rare tumor types exhibiting bronchiolar features according to their immunofluorescence (IF) staining, although it is difficult to distinguish them by their histological morphologies (Figure 1C,D). Using IF staining, we demonstrated that majority of the tumors expressed the AT2 cell marker prosurfactant protein C (proSPC), while rare tumors also had expression of club cell secretory protein (CCSP) or the bronchiolar transcription factor SOX2. In this model, cross‐sections showed between 0 and 10% of tumors in a given mouse had SOX2 expression (Figure S1A, Supporting Information) and both SOX2+ and SOX2‐ tumors expressed high levels of EGFR (Figure S1B, Supporting Information). The staining specificity was validated using *KrasG12D; p53‐null* tumors that had low EGFR expression in both SOX2+ and SOX2‐ regions (Figure S1C, Supporting Information). These results are similar to a previous study in *EGFR* TL mice, in which the tumors were termed as bronchial and peripheral.^[^ ^22^ ^]^

### 2.2 Distal Lung Stem/Progenitor Cells Efficiently Undergo Ex Vivo Malignant Transformation by Mutant EGFR

**Figure 2:**
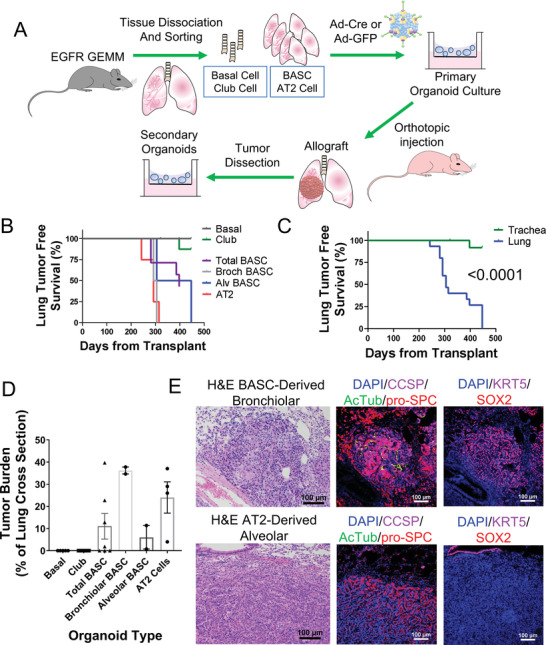
Distal lung stem/progenitor cells efficiently undergo ex vivo malignant transformation by mutant EGFR. A) Schematic of cell‐of‐origin study in lung stem or progenitor cells including FACS sorting,in vitro propagation and adenovirus activation of EGFR, in vivo orthotopic transplantation, and secondary in vitro culture. B,C) Tumor‐free survival of immunocompromised mice with the indicated transplanted organoid types, *p* < 0.0001 between organoids from distal lung (lung) and proximal lung (trachea) by Mantel–Cox log‐rank test. D) Tumor burden as percentage of total lung was analyzed by ImageJ for indicated transplanted organoid types, mean ± standard error of the mean (SEM) is graphed. E) IF analysis of orthotopic transformed bronchiolar and alveolar tumors stained with the indicated probes.

Next, in order to understand if there are different cells in the normal lung epithelium that can serve as cells‐of‐origin for the EGFR‐driven lung cancers, we used our in vitro transformation strategy.^[^ ^7^ ^]^ Lungs and tracheas from nontumor bearing mice were isolated and the cells were dissociated and sorted for nerve growth factor receptor (NGFR)+ basal cells of the trachea, NGFR‐ club cells of the trachea, Stem Cell Antigen 1 (Sca1/Ly6A)+ BASCs of the distal lung, and Sca1‐ AT2 cells of the distal lung by fluorescence‐activated cell sorting (FACS) (Figure S2A, Supporting Information). FACS‐isolated lung cells were then divided into two aliquots, one of which received Adeno‐green fluorescent protein (GFP) control virus, and the other one received Adeno‐Cre virus to activate mutant EGFR (**Figure** **2**A). The cell populations were then plated into 3D Matrigel organotypic cultures, which also contained neonatal lung mesenchymal endothelial cells as supporting cells.^[^ ^23^ ^]^ Organoids post viral infection were allowed to grow and then were passaged. Organoids from each cell population tolerated the activation of *EGFR* TL mutations and continued to grow. During passage, organoids derived from BASCs were manually subcloned into alveolar, bronchiolar, and mixed cultures by their microscopic morphologies. Mixed BASCs had both alveolar and bronchiolar organoids (Figure S2B, Supporting Information). Then, the four subtypes of organoids infected by Adeno‐Cre virus were orthotopically transplanted to the lungs of immunocompromised mice to allow for further in vivo transformation. The mice started to show signs of lung tumors 8 months post transplantation of AT2 cells or BASCs, including the bronchiolar, alveolar, and the mixed BASC organoids (Figure 2B). Only one mouse developed a small tumor nodule from tracheal club cell‐derived transplantation (Figure S2C, Supporting Information). None of the mice that received basal cell‐derived organoids had tumors at sacrifice. Due to these differences, mice transplanted with distal lung (BASC and AT2) organoids had a significantly shorter lung‐tumor free survival than the proximal lung/trachea (basal cell and club cell) organoid transplantation (*p* < 0.0001, Figure 2C). To validate that EGFR was activated in proximal cells, we performed reverse transcription polymerase chain reaction (RT‐PCR) for the human *EGFR* transcript and also checked gene expression of the knock‐in locus *Col1a1* and of the tumor suppressor *Trp53* (Figure S2D, Supporting Information). We found that EGFR transcript was induced in proximal lung cells similarly to distal and that *Col1a1* and *Trp53* expression were minimally changed by Cre virus. Bronchiolar BASCs and AT2 cells formed tumors with a higher tumor burden than the other organoid types, although high heterogeneity existed among each mouse (Figure 2D).

Next, we analyzed the histology of the transformed tumors to study whether the tumors maintained their original lineage properties during in vivo transformation. It showed that most of the BASCs and AT2 derived tumors formed alveolar tumors in vivo, with positive staining of alveolar marker SPC and barely any expression of bronchiolar markers CCSP and SOX2, or the basal cell marker KRT5 (Figure 2E). Bronchiolar BASCs formed a small number of orthotopic bronchiolar tumor nodules, although the majority of the other tumors displayed positive staining of alveolar lineage marker. It suggested that a large part of the bronchiolar BASCs underwent lineage switch to alveolar state during transformation, probably due to the impact of specific microenvironment in which it may be easier for alveolar cells to reside. Altogether, these results suggested that the AT2 cells and BASCs were the main cellular origins of EGFR mutant lung cancer and these tumors retained the innate properties of their derived stem or progenitor cells during the process of tumorigenesis.

### 2.3 Different Stem/Progenitor Cells Drive Distinct Gene Expression during Malignant Transformation

**Figure 3:**
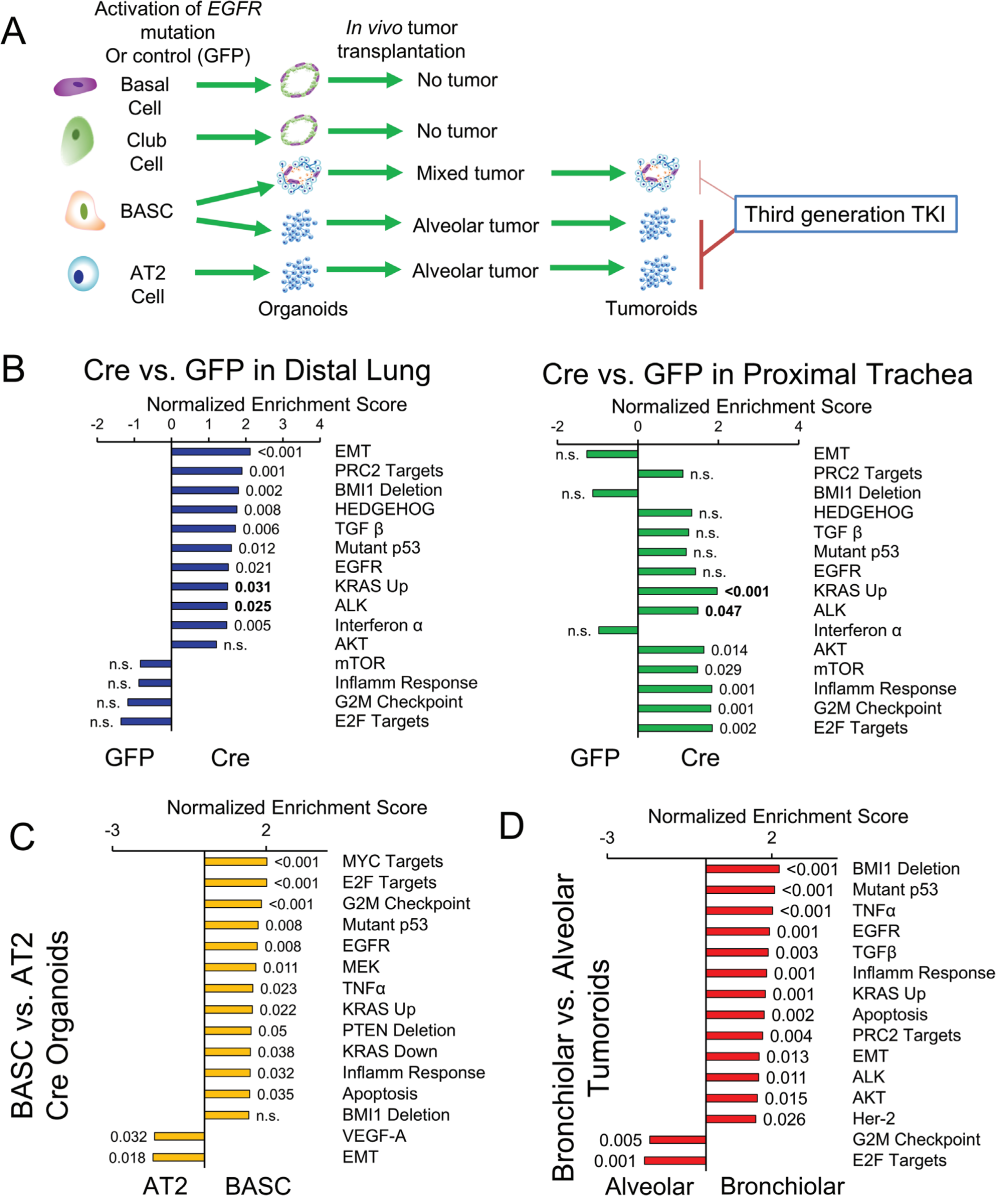
Different stem/progenitor cells drive distinct gene expression during malignant transformation. A) Schematic of RNA‐sequencing samples from organoids with indicated treatment. B) Bar plots of normalized enrichment scores of selected gene signatures enriched in Ad‐Cre treated distal lung (left) or proximal trachea (right) organoids relative to Ad‐GFP treated organoids, with false discovery rate (FDR) *q*‐values indicated outside the end of bars, *q* values in bold are pathways significant in both distal lung and trachea. C) Bar plots of normalized enrichment scores of selected gene signatures enriched in BASC‐derived organoids relative to AT2‐derived Ad‐Cre treated organoids, with FDR *q*‐values indicated outside the end of bars. D) Bar plots of normalized enrichment scores of selected gene signatures enriched in bronchiolar tumoroids relative to alveolar tumoroids, with FDR *q*‐values indicated outside the end of bars.

In order to understand the deeper mechanisms of tumorigenesis and progression induced by *EGFR* TL mutation, we performed RNA‐sequencing on the various stem/progenitor cell‐derived organoids with Ad‐GFP or Ad‐Cre virus and also tumor cells after orthotopic transplant (**Figure** **3**A). To make sure equivalent RNA samples were being compared, the transplanted tumors were dissociated and propagated in 3D Matrigel culture in vitro prior to RNA extraction. IF staining of the lineage markers showed that tumoroids from alveolar tumors highly expressed alveolar marker SPC with no or low expression of bronchiolar markers CCSP and SOX2, and the basal cell marker KRT5, while the bronchiolar tumoroids, which were only obtained from bronchiolar BASC‐derived transplants, expressed the opposite pattern (Figure S2A, Supporting Information). This result suggests that tumor cells retain their intrinsic lineage properties when propagating in vitro.

Hierarchical cluster analysis showed that post‐transplant tumoroids clustered together, away from the pretransplant organoids (Figure S2B, Supporting Information). Intriguingly, tumoroids from an in vivo autochthonous tumor were transcriptionally closer to mixed BASC‐derived and AT2‐derived tumoroids. To understand the transcriptional alterations that happened during the transformation by *EGFR* T790M/L858R mutation in vitro and in vivo, we compared the gene expression on the organoids with Ad‐GFP or Ad‐Cre virus infection and before or after transplantation. The gene set enrichment analysis (GSEA) demonstrated that activation of EGFR mutations in distal lung cells led to significant enrichment of genes associated with epithelial‐mesenchymal transition (EMT), Hedgehog signaling, transforming growth factor beta (TGF‐*β)*, KRAS, and also EGFR pathways. Activation of EGFR mutations in proximal lung/tracheal cells caused enriched hallmarks of KRAS, E2F targets, G2M checkpoints, MTORC1, and AKT serine/threonine kinase (AKT) pathways (Figure 3B). The EGFR pathway was enriched, but not significantly, in the proximal lung organoids after EGFR activation. Within the distal lung epithelia, BASC‐derived Ad‐Cre organoids generally had higher gene expression in MYC targets,E2F targets, interferon‐*α* response, G2M checkpoints, mutant p53, and EGFR pathways and lower expression of EMT and vascular endothelial growth factor‐A signatures than the AT2‐derived Ad‐Cre organoids (Figure 3C).

Then, to further study the tumorigenic mechanisms during orthotopic transformation, we compared BASC‐ and AT2‐derived tumoroids post‐transplant with pretransplant. Although the pre‐ and post‐transplant samples were sequenced at different platforms (see the Experimental Section),principal component analysis did not detect obvious confounding variables separating these two platforms. GSEA results then indicated that the gene sets relevant to MYC proto‐oncogene, bHLH transcription factor (MYC) targets, mechanistic target of rapamycin kinase (mTOR) signaling, DNA repair, and deletion of RB were higher in post‐transplant tumoroids than in pretransplant, while epithelial to mesenchymal transition (EMT), EGFR, and KRAS upregulated gene signatures were less enriched (Figure S2C, Supporting Information). Genes that decrease when embryonic ectoderm development (EED) or enhancer of zeste 2 polycomb repressive complex 2 subunit (EZH2) are depleted in human fibroblasts were upregulated in post‐transplant tumoroids, suggesting that during transplantation, expression or enzymatic function of Polycomb Repressive Complex 2 (PRC2) may be increased. To link this discovery to clinical samples, we investigated the gene signature changes between EGFR mutant tumor samples (including L858R, T790M mutations or exon 19 deletions) and normal lung tissues. Excitingly, out of the 15 gene signatures, 14 signatures were enriched in the same direction in both the mouse and human datasets, and 11 signatures were significantly enriched in the human dataset in the same direction as expected from the mouse experiment (Figure S2D, Supporting Information). Therefore, these signaling pathways regulated during the in vivo transformation may serve as potential therapeutic targets when combined with EGFR inhibition.

Furthermore, to interrogate the difference between bronchiolar and alveolar tumors, we then compared the transcriptional phenotypes of bronchiolar tumoroids derived from BASCs with alveolar tumoroids from either BASCs or AT2 cells according to the expression of their lineage markers. GSEA results demonstrated that bronchiolar tumoroids had transcriptional enrichment in TNF‐*α*, KRAS, mutated p53, EGFR, BMI1 deletion, and PRC2 activity associated pathways versus alveolar tumoroids (Figure 3D). Conversely, alveolar tumoroids expressed higher E2F targets and G2M checkpoint genes than bronchiolar tumoroids. Together, these data displayed that in vitro activation of *EGFR* T790M/L858R mutations and in vivo orthotopic transformation drove distinct transcriptional landscapes in cells with different cellular origins.

### 2.4 Bronchiolar and Alveolar Tumoroids Respond to Therapies Differentially

**Figure 4:**
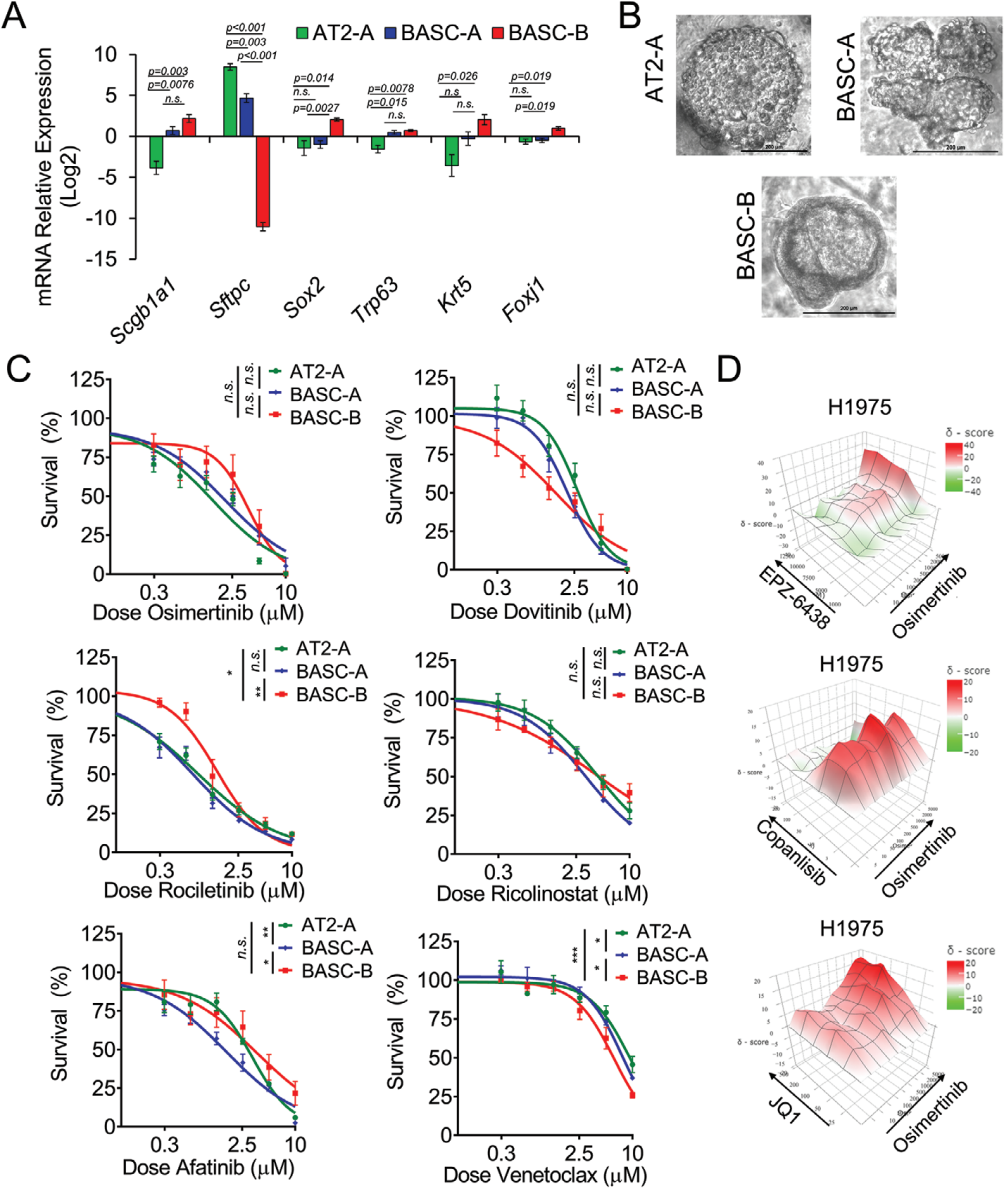
Bronchiolar and alveolar tumor organoids have distinct drug responses. A) Relative mRNA expression of lineage genes in the indicated mouse 3D tumoroids determined by RT‐qPCR, *n* = 4 experimental replicates for BASCs and *n* = 3 experimental replicates for AT2‐A, mean ± SEM is graphed. B) Representative bright field images of the indicated 3D tumoroids propagated in the transwells. C) Dose responses to drugs osimertinib, rociletinib, afatinib, dovitinib, ricolinostat, and venetoclax in the indicated tumoroids, *n* = 4 experiments. D) Heatmap of Bliss synergy scores of osimertinib combined with EPZ‐6438, copanlisib, and JQ1 in H1975 2D cultures with three separate experiments used to produce the final matrix. Overall Bliss scores with 95% confidence intervals were 1.36 ± 3.82 for osimertinib+EPZ‐6438, 3.36 ± 3.56 osimertinib+copanlisib, and 7.82 ± 2.49 osimertinib+JQ1. Most synergist area Bliss scores were 13.20 for osimertinib+EPZ6438, 9.06 for osimertinib+copanlisib, and 15.38 for osimertinib+JQ1.

Next, to interrogate whether tumors with different cell fates have distinct sensitivities to therapies, especially TKIs, we performed dose‐response assessment on the bronchiolar and alveolar 3D tumoroids. First, we confirmed the expression of lineage markers of these tumoroids by RT‐PCR. As expected, BASC‐derived bronchiolar (BASC‐B) tumoroids had significantly lower expression of alveolar marker *Sftpc*, higher expression of the club cell marker *Scgb1a1*, and higher expression of proximal lung epithelial markers including *Sox2*, *Trp63*, *Krt5*, and *Foxj1* than the AT2‐derived alveolar (AT2‐A) tumoroids (**Figure** **4**A). Tumoroids of alveolar lineages also had higher expression of human *EGFR* transgene, despite having similar activity of the *Col1a1* locus (Figure S4A, Supporting Information). To ensure that all tumoroids were derived from *EGFR*‐donor mice, PCR on genomic DNA was performed on tail samples (recipient mouse), tumor samples and two different passages of each tumoroid culture (Figure S4B, Supporting Information). The results show that BASC‐B tumoroids, despite having lower *EGFR* expression, are definitely derived from *EGFR* donor mice. Intriguingly, BASC‐derived alveolar (BASC‐A) tumoroids expressed intermediate levels of both *Scgb1a1* and *Sftpc*, and intermediate levels of the proximal airway lineage markers *Trp63* and *Krt5*. This suggested that BASC‐A cells may be in a mixed lineage state, keeping the dual‐potential to become alveolar and bronchiolar cells during passages. Consistently, BASC‐A contained morphologically bronchiolar, alveolar, and mixed tumoroids (Figure 4B).

Next, we tested first generation TKI erlotinib, second generation TKI afatinib, third generation TKIs osimertinib and rociletinib, pan‐receptor tyrosine kinase inhibitor dovitinib, HDAC6 inhibitor ricolinostat, and BCL2 apoptosis regulator inhibitor venetoclax on AT2‐A, BASC‐A, and BASC‐B tumoroids. Overall, tumoroids had lower IC_50_ values for third generation TKIs than the first and second generation TKIs (Figure S4C, Supporting Information). Interestingly, it showed that AT2‐A and BASC‐A tumoroids were significantly more sensitive to third generation TKI rociletinib than BASC‐B tumoroids in in vitro 3D culture (adjusted *p *= 0.02, and adjusted *p *= 0.0036, respectively, Figure 4C). BASC‐B tumoroids also had 2.5‐fold higher IC_50_ to osimertinib than AT2‐A. Similar results were observed in *EGFR TL‐*driven lung cancer previously, which the peripheral tumors were relatively more sensitive to HKI‐272, an irreversible EGFR inhibitor, than the bronchial tumors.^[^ ^22^ ^]^ No significant difference of IC_50_ values was observed in these tumoroids to afatinib. Expectedly, erlotinib did not manifest a potent therapeutic effect on these tumoroids with T790M mutation (Figure S4D, Supporting Information). On the contrary, BASC‐B were more responsive to dovitinib than the AT2‐A tumoroids. Venetoclax also harbored a lower IC_50_ in BASC‐B than BASC‐A and AT2‐A tumoroids (Figure 4C). Since the alveolar and bronchiolar tumoroids had the same mutations, these results suggested that different epigenetic states affected the drug vulnerabilities to TKIs and other therapies.

### 2.5 Combination Therapies Can Increase Osimertinib Response

Lastly, we aimed to detect potential therapies which could combine with and improve the therapeutic effect of TKIs in the relatively insensitive tumors. GSEA results comparing post‐transplant tumoroids with pretransplant organoids indicated that MYC, mTOR,and EZH2‐target pathways were enriched during the in vivo transformation. Therefore, we tested osimertinib combined with EZH2 inhibitor EPZ‐6438/tazemetostat, Bromodomain and Extra‐Terminal motif (BET) inhibitor JQ1, which was proved to suppress MYC function, or PI3K/AKT/mTOR pathway inhibitor copanlisib on *EGFR*‐mutant tumoroids and lung cancer lines.^[^ ^24^, ^25^, ^26^ ^]^ In the previous studies, EZH2 inhibition has been observed to reverse gefitinib resistance in EGFR‐wildtype lung cancer and synergize with HER2‐targeted breast cancer treatment but also showed conflicting results of conferring resistance to gefitinib in lung cancer.^[^ ^27^, ^28^, ^29^ ^]^ It has been reported that irreversible TKI synergized with the mTOR inhibitor rapamycin in peripheral and bronchial *EGFR TL* tumors.^[^ ^22^ ^]^ PI3K‐AKT‐mTOR inhibition was also reported to sensitize lung cancer cells to or overcome resistance to EGFR‐TKIs.^[^ ^30^, ^31^, ^32^, ^33^, ^34^, ^35^ ^]^ BET inhibition was reported to delay the acquired resistance to anti‐EGFR antibody in head and neck squamous cell carcinoma, and synergize with anti‐HER2 TKIs in lung cancer and breast cancer.^[^ ^36^, ^37^, ^38^ ^]^ The results showed that JQ1 and copanlisib combined with osimertinib led to significantly more potent inhibitory effect than using these drugs alone in BASC‐A tumoroids (Figure S4E, Supporting Information). EPZ‐6438 combined with osimertinib also induced a lower cellular survival than EPZ‐6438 alone, but not significantly lower than osimertinib alone. Then, we verified this synergistic effect in human cell lines PC9‐GR4 and H1975, which both possess the *EGFR T790M* mutation. H1975 also had *EGFR L858R* mutation. The synergy matrix of osimertinib combined with EPZ‐6438 displayed good synergist effect in PC9‐GR4 and H1975, while the osimertinib combined with copanlisib was additive in H1975, with marked synergism between 3 × 10^−9^ and 10 × 10^−9^ m copanlisib dosing, and synergistic in PC9‐GR4 (Figure 4D and Figure S4F, Supporting Information). JQ1 also had synergy with osimertinib in H1975 but not in PC9‐GR4, suggesting that the PC9‐GR4 cell line possibly harbors mutations antagonistic toward BET inhibition. Together, these data shed light on promising therapeutic combinations with third generation TKIs in *EGFR* mutant lung cancers.

## 3 Conclusion and Future Work

It is well known that EGFR activating mutations are predominant in lung adenocarcinoma but not in lung squamous cell carcinoma.^[^ ^39^ ^]^ In our model system, regardless of the cell‐of‐origin, the majority of the transplanted and autochthonous tumors expressed the alveolar type 2 cell marker surfactant protein C. Even EGFR‐activated bronchiolar BASCs, when transplanted, formed mainly alveolar tumors. In the similar EGFR TL mouse model driven by a *TetO* promoter, the bronchiolar tumors were readily observed in all four of the founder mice, and this is in contrast to the more rare SOX2+ tumors we observed in only four out of ten mice examined.^[^ ^22^ ^]^ The reason behind the difference might be because in our mouse model the EGFR TL mutations were inserted into the *collagen1a1* locus. The differentially expressed genes of RNA‐sequencing data indicated that genes related to various types of collagen proteins, including *Col1a1*, were more frequently expressed in the alveolar cells than the bronchiolar cells, possibly indicating a slight preference for oncogenic EGFR to be more highly expressed in alveolar lung cells. Also, interestingly, the tumor burdens of bronchiolar‐derived tumors were larger than the alveolar‐derived tumors. It is known that both club cells and BASCs can give rise to alveolar cells.^[^ ^3^, ^40^, ^41^ ^]^ Research demonstrated that AT2 cells might not be the major contributor of the regeneration of lung epithelium after bleomycin injury, while CCSP‐expressing cells may play a significant role in this process.^[^ ^3^, ^42^, ^43^, ^44^ ^]^ These data suggest that BASCs possess a potent potential for alveolar cell differentiation and could serve as cells‐of‐origin for diverse tumor types.

We also discovered that basal and club cells from the mouse trachea were unable to yield intratracheally transplantable organoids when EGFR TL mutation was activated ex vivo. As lung squamous cell carcinoma is proposed to originated from basal cells, it implied that EGFR‐mutant squamous cell carcinomas might be derived from trans‐differentiation of lung adenocarcinoma.^[^ ^4^ ^]^ However, the mechanism of why basal and club cells could not develop in vivo tumors needs further study. It might be because EGFR TL mutation drove mainly alveolar tumors, the cell type to which basal and club cells could not easily give rise. Another possibility is that trachea‐derived cells lack the ability to engraft in the intratracheal instillation model that we employed. Lastly, according to the GSEA results, the basal and club cells did not significantly upregulate the EGFR signaling pathway when EGFR TL mutations were activated by adeno‐Cre virus, which likely reflects a different response to the oncogene that may explain the lack of engraftment of these cells.

The dose curve assays showed that bronchiolar and alveolar tumors respond differentially to TKIs and other therapies. Especially, the third generation TKI osimertinib and rociletinib targeting T790M gatekeeper mutation were both more effective in the AT2‐derived alveolar tumoroids than the BASC‐derived bronchiolar tumoroids. One possible theory is that bronchiolar and alveolar cells possess different compositions of EGFR homo‐ and heterodimers, so that they have different sensitivities to reversible, irreversible, and mutant‐selective TKIs.^[^ ^22^ ^]^ Gene expression suggests that *EGFR* is less expressed in bronchiolar tumoroids; however, the EGFR signaling pathway was more enriched in the bronchiolar tumoroids than the alveolar ones. Therefore, the bronchiolar cells might require higher dose of osimertinib and rociletinib to achieve the same level of EGFR repression. The result may also be intrinsic to the SOX2+ nature of the bronchiolar organoids, as research in human cell lines has suggested SOX2 plays a role in osimertinib resistance.^[^ ^45^ ^]^ However, the clinical relevance of this finding will require further investigation, including in vivo experiments testing the response of each tumor lineage to systemic therapy.

## 4 Experimental Section

### Cell Culture

Human cell lines H1975 and PC9‐GR4 were propagated in Roswell Park Memorial Institute 1640 media (RPMI 1640 media) (Gibco, #11875‐093) with 8–9% fetal bovine serum (VWR), glutaMAX (Gibco, #35050‐079), and penicillin/streptomycin (Gibco #15140‐122) at 37 °C, 5% CO_2_. Both lines were authenticated by IDEXX CellCheck9. Mouse sorted lung organoids or dissociated tumor cells were resuspended in Dulbecco's Modified Eagle Medium/Nutrient Mixture F‐12 media (DMEM/F12 media) (Gibco #11330‐057) containing penicillin/streptomycin, glutaMAX, 8–9% fetal bovine serum and 0.9x Insulin/transferrin/selenium mixture (Corning or Gibco), mixed with growth factor‐reduced Matrigel (Corning) and low passage neonatal lung mesenchymal support cells, and pipetted into a 24‐well 0.4 µm Transwell insert (Corning, #CLS3470). Plasmocin was added into cultures regularly. To passage organoids or assess cell viability, media in the lower chamber was aspirated and 100–150 µL of Dispase (Corning, #42613‐33‐2) was added to the disc of Matrigel to be liquefied. After Matrigel dissolved, organoids were collected and trypsinized for passages or aliquoted to 96‐well black plates for 3D CellTiter‐Glo assays.

### Histology and Immunofluorescence Staining

Mouse tumors or 3D organoids were collected and fixed in 10% neutral‐buffered formalin overnight at room temperature and then transferred to 70% ethanol, embedded in paraffin and sectioned. 3D organoids were immobilized in Histogel before embedding and sectioning. Hematoxylin and eosin stains were performed in the Biospecimen Procurement and Translational Pathology Shared Resource Facility (BPTP SRF) of the University of Kentucky Markey Cancer Center. Images of H&E stained slides were scanned with an Aperio slide scanner, white space and trachea/esophagus/lymph nodes masked, and then were auto‐contrasted and saved as grayscale images. Then the tumor burden (ratio of tumors to the whole lung lobes) was calculated in ImageJ software. Immunofluorescence staining were done as described previously.^[^ ^23^ ^]^ The slides of tumors or organoids were immunostained with primary antibodies for CCSP (Millipore ABS1673), pro‐SPC (Millipore ABS3786), Acetylated‐tubulin (SIGMA T7451), KRT5 (Biolegend PRB‐160p), EGFR (Bethyl IHC‐00005) and SOX2 (R&D Systems AF2018), and secondary antibodies donkey antimouse Alexa Fluor 488, antigoat Alexa Fluor 488, antirabbit Alexa Fluor 594, antigoat Alexa Fluor 594, antirabbit Alexa Fluor 647 and antigoat Alexa Fluor 647, and mounted with ProLong Gold Mounting media with 4′,6‐diamidino‐2‐phenylindole (DAPI). Fluorescence images were captured on Nikon Ti‐Eclipse inverted microscope. Exposures and look‐up‐tables were matched for all images of each stain at each magnification, with the exception of DAPI look‐up‐tables that were set for best visualization.

### Quantitative RT‐PCR, RNA‐Sequencing, and GSEA

RNAs from 3D organoids were isolated using Absolutely RNA kits (Agilent) and cDNAs were made with the SuperScript III (Invitrogen). Relative gene expression of lineage markers was detected with Taqman probes on Quant Studio 3 Real‐Time PCR system. Relative expression of genes of interest was calculated by (Ct_ref_ − Ct_exp_) where the reference Ct is the average of all Ct values for the gene of interest from all replicates and samples, then normalized the multiplexed house‐keeping gene *Gapdh* and graphed on the log2 scale (Figure 4A and Figure S4A, Supporting Information) or to one experimental replicate of the GFP‐infected cells (Figure S2D, Supporting Information). One organoid sample was excluded due to very high *Gapdh C*_t_ values that indicated degraded sample. Statistics were performed on log2 data by two‐tailed Student's *T*‐test and corrected for multiple comparisons by Holm–Sidak *p*‐value adjustment for each gene. Probes were: *EGFR* Hs01076092_m1, *Col1a1* Mm00801666_g1, *Trp53* Mm01731290_g1, *Scgb1a1* Mm00442046_m1, *Sftpc* Mm00488144_m1, *Sox2* Mm03053810_s1, *Trp63* Mm00495793_m1, *Krt5* Mm01305291_g1, *Foxj1* Mm01267279_m1, and *Gapdh* endogenous control 4352339E. RNA samples from organoids were also sent for sequencing in the University of Kentucky Oncogenomics Shared Resource Facility (for pretransplant organoids) or by Beijing Genomics Institute (for post‐transplant tumoroids). Sequencing reads were trimmed and filtered using Trimmomatic (V0.39) to remove adapters and low quality reads.^[^ ^48^ ^]^ Reads of mouse samples were mapped to Ensembl GRCm38 transcripts annotation and normalized by RNA‐Seq by Expectation‐Maximization (RSEM) and R package edgeR.^[^ ^49^, ^50^ ^]^ Gene set enrichment analysis was performed with GSEA version 4.1.0 with gene expression vsd‐normalized by DESeq2 R package.^[^ ^51^, ^52^ ^]^ Mouse genes were mapped to human orthologs by Mouse‐ENSEMBL_Gene_ID_to_Human_Orthologs_MSIGDB.v7.4 chip and queried for Hallmarks (h.all.v7.4) and Oncogenic signatures (c6.all.v7.4) (Table S1, Supporting Information). Results were graphed by bar plots using normalized enrichment scores and false discovery rate (FDR)*q*‐values (*q < 0.05* as significant difference). Human genes from the caner genome atlas (TCGA) were mapped to Human_ENSEMBL_Gene_ID_MSigDB.v7.4.chip. To examine pathway changes in the EGFR mutant lung cancer, gene expression data were analyzed from TCGA lung adenocarcinoma database.^[^ ^46^ ^]^ The gene expression raw counts and somatic mutation data were downloaded using TCGAbiolinks including 230 lung adenocarcinoma and 59 normal samples.^[^ ^47^ ^]^ In all, 18 EGFR mutant tumors with L858R, T790M mutations, or exon 19 deletions were identified. GSEA was performed comparing the EGFR mutant patient tumors (*n* = 18) versus normal patient samples (*n* = 59).

### Flow Cytometry Analysis and Sorting

Single cell suspensions of murine lung and tracheal cells were stained using rat‐antimouse antibodies including antimouse‐EpCAM‐PECy7 (BioLegend), antimouse‐CD31‐APC (BioLegend), and antimouse‐CD45‐APC (BioLegend), anti‐NGFR (AbCAM primary, antirabbit‐PE secondary), and anti‐Sca1/Ly6A (BioLegend). Live cells were gated by exclusion of DAPI positive cells (Sigma, #D9542). All antibodies were incubated for 10–15 min at 1:100 dilutions. Cell sorting was performed with a Sony iCyt with a 100 µm nozzle, and the sorted cells were collected for further 3D culture. Cell infections were performed as described.^[^ ^7^ ^]^ Briefly, sorted cells were incubated in 100 µL of 6 × 10^7^ pfu mL^−1^ adeno‐Cre or adeno‐GFP virus (University of Iowa) in complex media for 1–2 h at 37 °C, 5% CO_2_. Cells were then washed by pelleting and resuspending prior to being plated in transwells with Matrigel and low passage neonatal lung mesenchymal support cells.

### Animal Work

All care and treatment of experimental animals were approved by the University of Kentucky Institutional Animal Care and Use Committee guidelines. Cohorts of both male and female mice were used for autochthonous tumor experiments, and no sex differences were noted. Adult mice received 2.9 ×10^7^ PFU adeno‐Cre virus (University of Iowa). Immunocompromised mice (Nude, *Foxn1* ^Nu/Nu^) were used for orthotopic intratracheal instillation with AdenoCre‐treated organoids. Mice at endpoint due to high tumor burden were used for the overall survival graph. Each mouse received ≈1.3 × 10^5^ dissociated organoid cells by inhalation of cells through catheter inserted in trachea. Six nude mice were excluded from final analysis due to development of lymphoma or early unexplained death. Tumors and resulting organoids were confirmed to be derived from donor mouse cells by genomic DNA PCR using 150 ng of template DNA and the following primer pairs: Control Locus (*Ezh2*) F: 5’‐CCCATGTTTAAGGGCATAGTGACATG‐3’ Control Locus (*Ezh2*) R: 5’‐ATGTGC AGGTCAGTCAGCAACTTCAG‐3’ Human *EGFR* knock‐in F: 5’‐CCCGTCGCTATCAAGGAATTA‐3’ Human *EGFR* knock‐in R: 5’‐GACATCACTCTGGTGGGTATAG‐3’. PCRs were visualized on 2% agarose gels with 100 bp DNA ladder (Invitrogen).

### 3D Cell Viability Dose Response Assay

The EGFR organoids previously maintained in transwells of 24‐well plates were disposed, washed, digested with trypsin. Cells were seeded in 20 µL of Matrigel to 384‐well Corning Spheroid microplates at 200–300 cells per well together with 5000 cells per well of mouse endothelial cells. A 10 µL of medium was applied to the top of Matrigel. After culturing for 3 d when there appeared noticeable organoids under microscope, 20 µL of drug solutions were added to the wells. After treatment for 3 d, cell viability was measured with CellTiter‐Glo 3D (Promega). Briefly, 25 µL of GellTiter‐Glo 3D lysis buffer was added to each well. After being racked on a platform for 5 min and incubated at room temperature for 25 min, the luminescence signal was measured in Cytation5 luminometer. Values were normalized to vehicle controls for each drug's columns to yield percent survival, and drug doses converted to a log scale for graphing as log(inhibitor) versus response − variable slope (four parameters). Bottoms were constrained to zero and vehicle wells were set to 1E‐10. P values comparing IC_50_ values were extracted and corrected with Holm–Sidak *p* value adjustment for each drug.

### Synergy Assay

For synergy assays, adherent cells were plated in 96 well format and treated with a matrix of drug doses, and at 4 d, CellTiter Glo was used to measure cells in each well. The percentage survival for drug combinations for three independent replicate experiments were input as replicates to https://synergyfinder.fimm.fi/ to calculate Bliss synergy scores (readout: viability; baseline correction: yes) and display 3D a synergy heatmap (>1 means additive response, >10 means synergism).^[^ ^53^ ^]^

### Statistics and Reproducibility

All graphed data were presented as mean ± standard error of the mean (s.e.m.) unless otherwise noted. For RT‐qPCR, dose response assays and synergy matrix, 3‐6 independent replicate experiments were used to generate averaged data. Exact *n* are indicated in figure legends. Exact data handling for RT‐qPCR and dose response assays are described in their experimental sections. Unless indicated, *p* values represent 2‐tailed Student's *t*‐test with equal variance that were used to compare continuous outcomes between two experimental groups and data passed a Shapiro‐Wilk test for normality, with the exception EGFR transcript data for BASC‐B of Figure S4A for which a Mann Whitney U test was used. Kaplan‐Meier curves and Mantel Cox log‐rank tests were used for survival outcomes. A p value less than 0.05 was considered statistically significant, and Holm‐Sidak adjusted p‐values are shown for Figures 4A,C and Supplementary Figure 4A. Statistical analyses were carried out using Excel or GraphPad Prism.

## Author Contributions

The author contribution is as follows: conceptualization (F.C., C.W., J.L., H.N.B.M., R.M.F., and C.F.B.); data curation (J.L., F.C., C.F.B., and C.W.); formal analysis (F.C., J.L., R.M.F., and C.F.B.); funding acquisition (H.N.B.M., C.W., and C.F.B.); data acquisition and processing (F.C., X.S., A.R.E., K.J.N., A.L., and C.F.B.); generation of the EGFR TL conditional mouse model (H.Z. and K.‐K.W.); bioinformatics and biostatistics (R.M.F., H.N.B.M., J.L., C.W.); writing—original draft (F.C. and C.F.B.); Writing—review and editing (F.C., R.M.F., H.N.B.M., and C.F.B.). We also thank D. Gilbreath in the Markey Cancer Center Research Communications Office for graphic designs.

## Conflict of Interest

The authors declare no conflict of interest.

## Supporting information

Supporting Information

## Data Availability

The RNA‐sequencing data for study are available at the National Center for Biotechnology Information Gene Expression Omnibus (NCBI GEO) database under GSE180360. The data that support the findings of this study are available from the corresponding author upon reasonable request.
