# Supplementary material for: Cellular Origins of EGFR‐Driven Lung Cancer Cells Determine Sensitivity to Therapy
Source: Adv Sci (Weinh). 2021 Oct 7;8(22):2101999. doi: 10.1002/advs.202101999 (PMC8596110; doi:10.1002/advs.202101999)
Supplement: Supplementary file 1 — Supporting Information [file ADVS-8-2101999-s001.pdf]

© 2021 Wiley-VCH GmbH

ADVANCED SCIENCE
Open Access

Supporting Information

for *Adv. Sci.*, DOI: 10.1002/advs.202101999

Cellular Origins of EGFR-driven Lung Cancer Cells Determine Sensitivity to Therapy

*Fan Chen, Jinpeng Liu, Robert M. Flight, Kassandra J. Naughton, Alexsandr Lukyanchuk, Abigail R. Edgin, Xiulong Song, Haikuo Zhang, Kwok-Kin Wong, Hunter N.B. Moseley, Chi Wang, and Christine F. Brainson**

Cellular Origins of EGFR-driven Lung Cancer Cells Determine Sensitivity to Therapy

Fan Chen[1], Jinpeng Liu[2], Robert M. Flight[3,4], Kassandra J. Naughton[1], Alexsandr Lukyanchuk[1], Abigail R. Edgin[1], Xiulong Song[1], Haikuo Zhang[5], Kwok-Kin Wong[6], Hunter N.B. Moseley[3,4], Chi Wang[2,4], Christine F. Brainson[1,4,#]

[1] Department of Toxicology and Cancer Biology, University of Kentucky, Lexington KY 40536 USA

[2]Department of Internal Medicine, University of Kentucky, Lexington KY 40536 USA

[3]Department of Molecular & Cellular Biochemistry, University of Kentucky, Lexington KY 40536 USA

[4] Markey Cancer Center, University of Kentucky, Lexington KY 40536 USA

[5]DNAtrix, 10355 Science Center Drive, Suite 110, San Diego CA 92121 USA

[6]Laura and Isaac Perlmutter Cancer Center, NYU Langone Medical Center, New York University, New York, NY 10016 USA

[#] Corresponding author

Christine Fillmore Brainson

Supplementary Figures 1-4

Supplementary Table 1

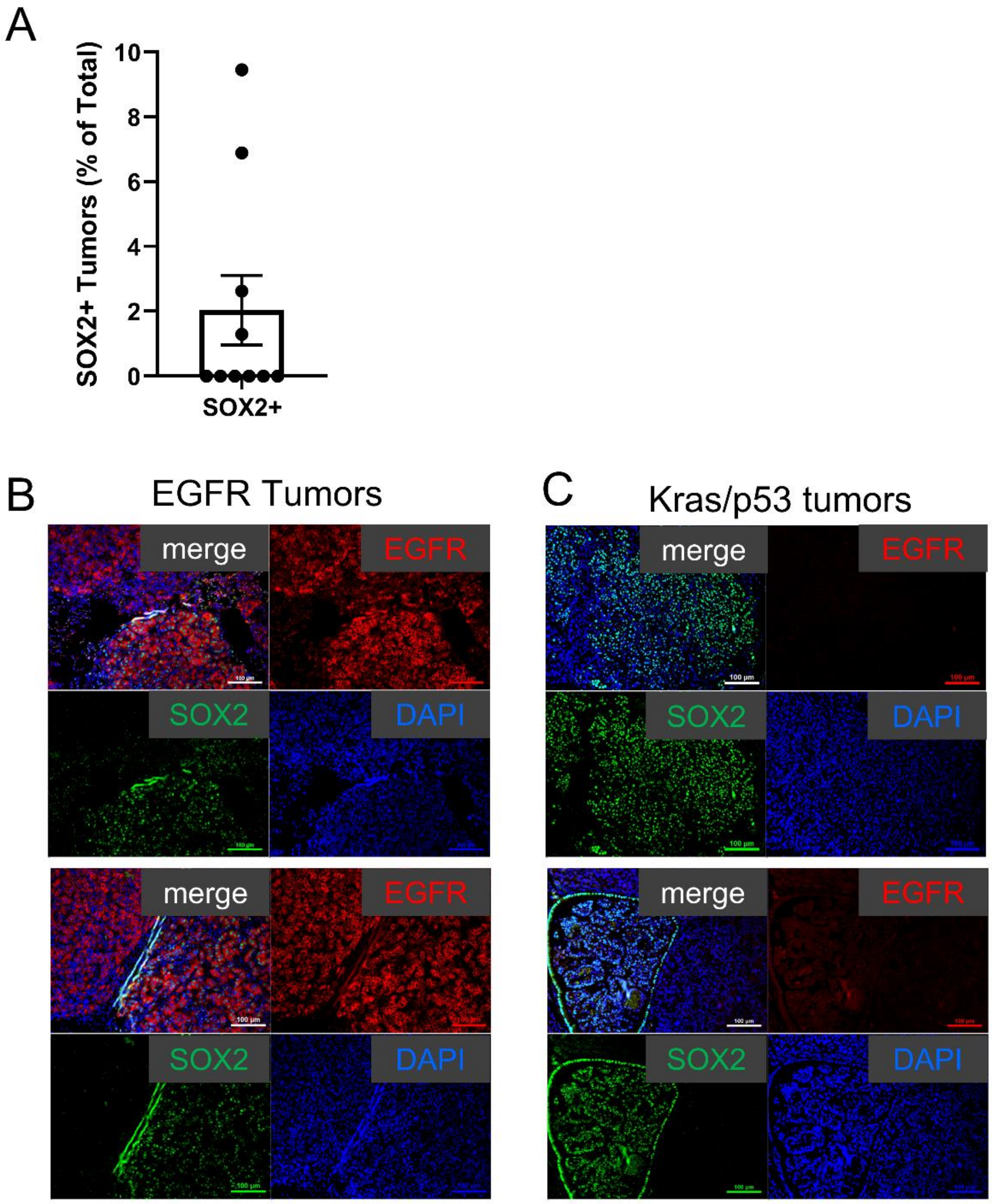

Supplementary Figure 1: The autochthonous LSL:EGFR T790M/L858R model develops lung adenocarcinoma in mice

(A) Graph of percentage of tumors in cross-sections that stained positively for SOX2. Mean ± SEM is graphed, n=10 mice. (B) Representative immunofluorescence staining of EGFR T790M/L858R autochthonous lung tumors with the indicated probes. (C) Representative immunofluorescence staining of *KrasG12D; p53-null* autochthonous lung tumors with the indicated probes.

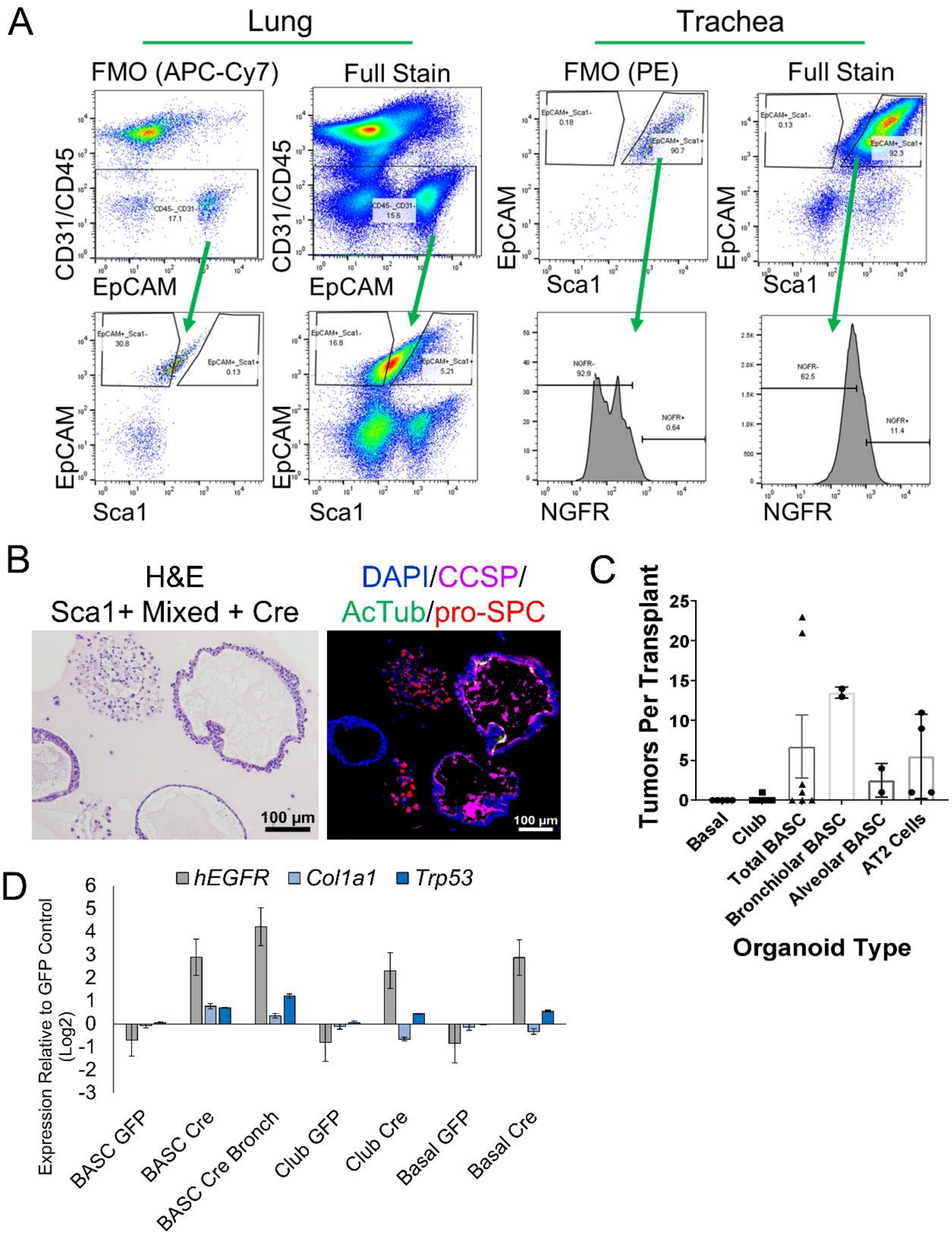

Supplementary Figure 2: Distal lung stem/progenitor cells efficiently undergo *ex vivo* malignant transformation by mutant EGFR (A) Schematic of sorted for NGFR+ basal cells of the trachea, NGFR- club cells of the trachea, Sca1/Ly6A+ BASCs of the distal lung and Sca1- AT2 cells of the distal lung. (B) H&E and IF analysis of BASC-derived mixed organoids stained with the antibodies against the indicated proteins. (C) Tumor counts from each mouse with

indicated organoid types transplanted, mean ± SEM is graphed. (D) mRNA expression of human *EGFR*, *Col1a1* and *Trp53* are graphed relative to the expression in matched GFP-control cells, n=1 biological replicate, n=2 independent qPCR experiments, mean ± SEM is graphed.

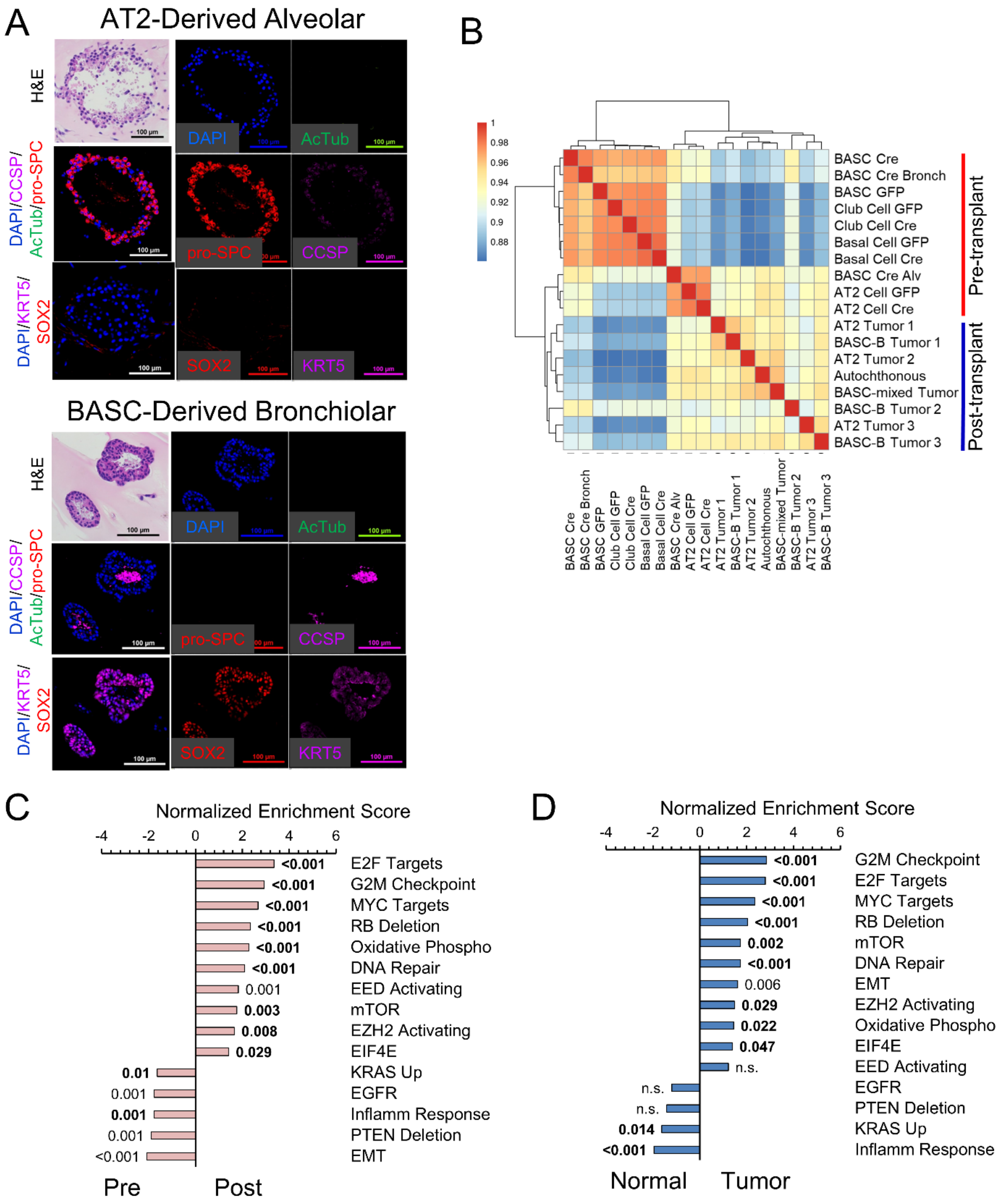

Supplementary Figure 3: Different stem/progenitor cells drive distinct gene expression during malignant transformation

(A) H&E and IF analysis of AT2-derived alveolar and BASC-derived bronchiolar tumoroids after transplant stained with the indicated probes. (B) Hierarchical clustering and dendsort plot using

principal component analysis 1 (PC1) of the variability of gene expression of the indicated samples. (C) Bar plots of normalized enrichment scores of selected gene signatures enriched in tumoroids after transplant relative to Ad-Cre treated organoids before transplant, with FDR *q*-values indicated outside the end of bars. (D) Bar plots of normalized enrichment scores of selected gene signatures enriched in EGFR mutant lung cancer tumors relative to normal lung tissues, with FDR *q*-values indicated outside the end of bars, *q* values in bold are pathways significant in both panels (C) and (D).

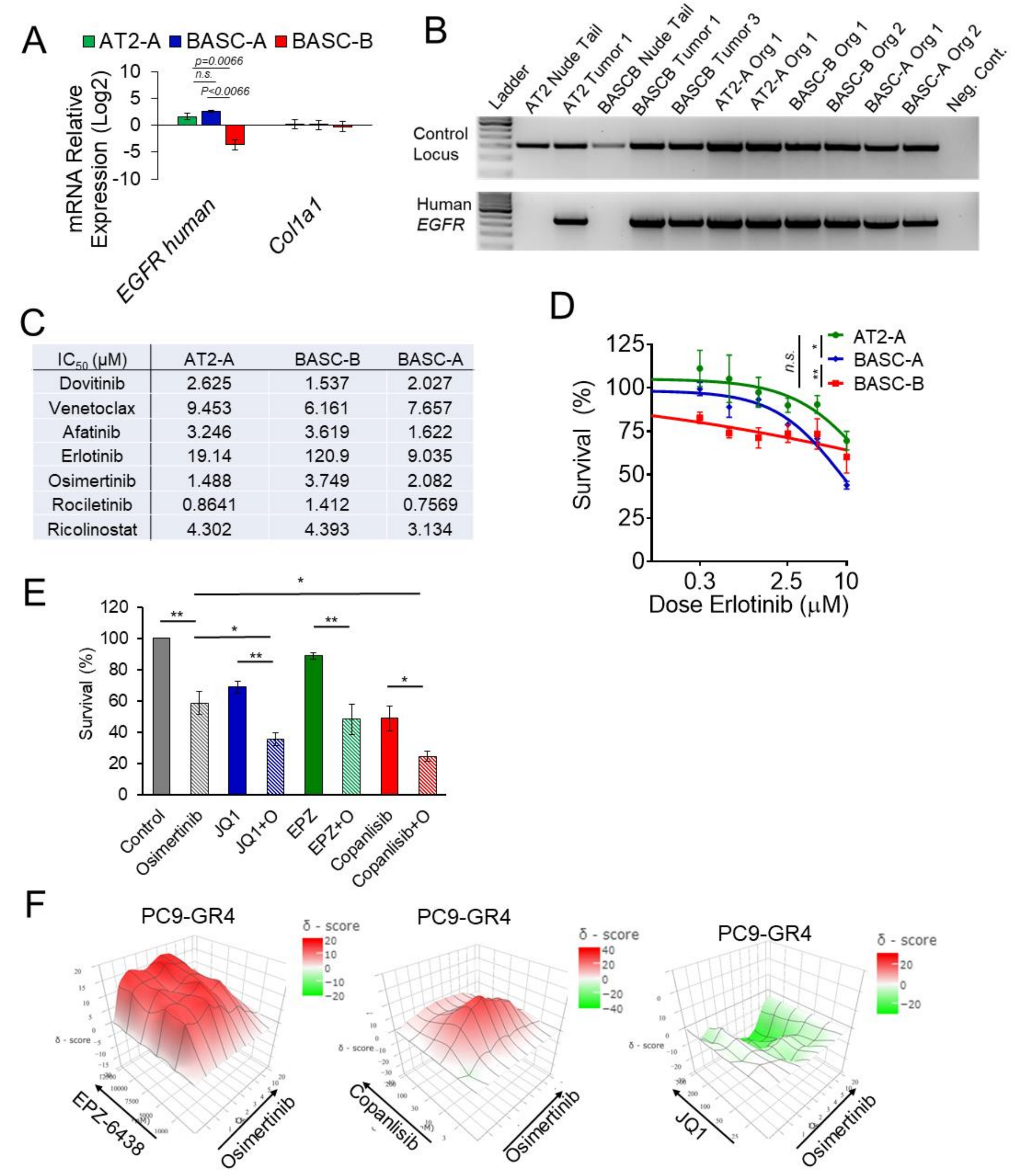

IC_{50} (μM)	AT2-A	BASC-B	BASC-A
Dovitinib	2.625	1.537	2.027
Venetoclax	9.453	6.161	7.657
Afatinib	3.246	3.619	1.622
Erlotinib	19.14	120.9	9.035
Osimertinib	1.488	3.749	2.082
Rociletinib	0.8641	1.412	0.7569
Ricolinostat	4.302	4.393	3.134

Supplementary Figure 4: Bronchiolar and alveolar tumor organoids have distinct drug responses (A) Relative mRNA expression of human *EGFR* and *Col1a1* in the indicated mouse 3D tumoroids determined by RT-qPCR, n=6 experimental replicates for BASCs and n=5 experimental replicates for AT2-A, mean +/- SEM is graphed. (B) PCR of genomic DNA show that all tumors and organoids are derived from EGFR-donor mice. (C) Averaged IC_{50} of the

indicated drugs calculated by the log(inhibitor) versus response curves. (D) Dose responses to drugs erlotinib in the indicated tumoroids, n=4 experiments. (E) Percentage of survival of BASC-A tumoroids with the indicated treatments of osimertinib 2.5 μM, EPZ-6438 5 μM, JQ1 50 nM and copanlisib 10 nM compared to control, mean ± SEM, n=4 for all except copanlisib n=3 individual experiments. (F) Heatmap of Bliss synergy scores of osimertinib combined with EPZ-6438, copanlisib, and JQ1 in PC9-GR4 2D cultures with 3 separate experiments used to produce the final matrix. Bliss scores with 95% confidence intervals were 11.6+/-2.6 for osimertinib+EPZ-6438, 9.18+/-1.79 osimertinib+copanlisib, and -9.1+/-1.43 osimertinib+JQ1. Most synergist area Bliss scores were 16.08 for osimertinib+EPZ6438, 17.15 for osimertinib+copanlisib, and -0.43 for osimertinib+JQ1.

Supplementary Table 1

MSigDB Gene Set Name	Descriptive Name
HALLMARK_G2M_CHECKPOINT	G2M Checkpoint
HALLMARK_E2F_TARGETS	E2F Targets
HALLMARK_MYC_TARGETS_V1	MYC Targets
RB_P107_DN.V1_UP	RB Deletion
HALLMARK_MTORC1_SIGNALING	mTOR
HALLMARK_DNA_REPAIR	DNA Repair
HALLMARK_EPITHELIAL_MESENCHYMAL_TRANSITION	EMT
PRC2_EZH2_UP.V1_DN	EZH2 Activating
HALLMARK_OXIDATIVE_PHOSPHORYLATION	Oxidative Phospho
EIF4E_UP	EIF4E
PRC2_EED_UP.V1_DN	EED Activating
EGFR_UP.V1_UP	EGFR
PTEN_DN.V2_UP	PTEN Deletion (Figure 3C)
PTEN_DN.V1_UP	PTEN Deletion (Figures S3 C-D)
HALLMARK_KRAS_SIGNALING_UP	KRAS Up
HALLMARK_INFLAMMATORY_RESPONSE	Inflamm Response
P53_DN.V1_UP	Mutant p53
MEK_UP.V1_UP	MEK
HALLMARK_TNFA_SIGNALING_VIA_NFKB	TNFα
HALLMARK_KRAS_SIGNALING_DN	KRAS Down
HALLMARK_APOPTOSIS	Apoptosis
BMI1_DN.V1_UP	BMI1 Deletion
VEGF_A_UP.V1_UP	VEGF-A
TGFB_UP.V1_UP	TGFβ
PRC2_EZH2_UP.V1_UP	PRC2 Targets
ALK_DN.V1_UP	ALK
AKT_UP.V1_UP	AKT
ERBB2_UP.V1_UP	Her-2
HALLMARK_HEDGEHOG_SIGNALING	HEDGEHOG
HALLMARK_INTERFERON_ALPHA_RESPONSE	Interferon α
